# Effect of oat or rice flour on pulse-induced gastrointestinal symptoms and breath hydrogen in subjects sensitive to pulses and controls – a randomised cross-over trial with two parallel groups

**DOI:** 10.1017/S0007114522000332

**Published:** 2022-12-14

**Authors:** Salla Laito, Niina Valkonen, Oskar Laaksonen, Marko Kalliomäki, Tuula Tuure, Kaisa M. Linderborg

**Affiliations:** 1 Food Chemistry and Food Development, Department of Life Technologies, University of Turku, Finland; 2 Valio Ltd, Helsinki, Finland; 3 Department of Pediatrics, University of Turku, Turku, Finland; 4 Department of Pediatrics and Adolescent Medicine, Turku University Hospital, Turku, Finland

**Keywords:** Breath hydrogen, Oats, Pulses, Gastrointestinal symptoms

## Abstract

Pulses are healthy and sustainable but induce gut symptoms in people with a sensitive gut. Oats, on the contrary, have no fermentable oligo- di-, monosaccharides and polyols compounds and are known for the health effects of their fibres. This 4-day cross-over trial investigated the effects of oat and rice flour ingested with pulses on gut symptoms and exhaled gases (4th day only) in subjects with a sensitive gut or IBS (*n* 21) and controls (*n* 21). The sensitive group perceived more symptoms after both meals than controls (*P* = 0·001, *P* = 0·001). Frequency, intensity or quality of the symptoms did not differ between meals during the first 3 d in either group. More breath hydrogen was produced after an oat than rice containing meal in both groups (AUC, *P* = 0·001, *P* = 0·001). No between-group difference was seen in breath gases. During day 4, both sensitive and control groups perceived more symptoms after the oat flour meal (*P* = 0·001, *P* = 0·0104, respectively) as mainly mild flatulence. No difference in moderate or severe symptoms was detected. Increased hydrogen production correlated to a higher amount of perceived flatulence after the oat flour meal in both the sensitive and the control groups (*P* = 0·042, *P* = 0·003, respectively). In summary, ingestion of oat flour with pulses increases breath hydrogen levels compared with rice flour, but gastrointestinal symptoms of subjects sensitive to pulses were not explained by breath hydrogen levels. Additionally, consumer mindsets towards pulse consumption and pulse-related gut symptoms were assessed by an online survey, which implied that perceived gut symptoms hinder the use of pulses in sensitive subjects.

Pulses are nutritionally valuable, sustainable and inexpensive foods. Globally, they are considered as the second most important food source after cereals^(1)^. An increased consumption of pulses is encouraged, for example, in the Nordic and Finnish Nutrition recommendations especially instead of red and processed meat (The National Nutrition Council of Finland 2014, Nordic Council of Ministers 2012). However, the consumption of pulses is known to induce gastrointestinal discomfort among subjects with irritable bowel syndrome, IBS^(2,3)^ and even in healthy subjects^(4,5)^. The galacto-oligosaccharides in pulses have been postulated to be the compounds triggering the perceived gastrointestinal symptoms (Tuck *et al.*, 2018). For example, in fava beans, approximately 10 % of fibres are*α*-galacto-oligosaccharides being mainly verbascose^(6)^.

Some patients with IBS have found that a low-FODMAP (fermentable oligo- di-, monosaccharides and polyols) diet relieves their symptoms^(7–9)^. The low-FODMAP diet excludes the consumption of pulses; vegetables; fruits and cereals, such as rye and wheat, from the diet due to their content of readily fermentable fibres. Oats, on the contrary, do not contain FODMAP compounds^(10)^ and thus might be more tolerated by individuals with IBS^(11)^ or in individuals with a ‘sensitive gut,’ i.e. those with undiagnosed gastrointestinal symptoms related to the consumption of foods containing FODMAP^(12)^. The EU has accepted a health claim that oat fibre increases fecal bulk^(13)^, which refers to improved bowel function. Moreover, the EU has accepted health claims on oat *β*-glucan for reducing blood cholesterol and postprandial glycaemia^(14)^. These effects are related to the increase of the viscosity of the digesta in the gastrointestinal tract and the ability of *β*-glucan chains to trap nutrients and decelerate their absorption^(15–17)^. Moreover, the increase of viscosity is known to reduce the accessibility of carbohydrates for bacteria and enzymes in the colon, which may decrease the fermentation rate^(18)^. In the long run, the consumption of *β*-glucan is known to be beneficial for the gut microbiota^(19,20)^, which has been implied to be compromised in the patients with IBS^(21,22)^.

Intestinal fermentation of carbohydrates is related to exhaled hydrogen and methane^(23)^. Earlier studies have detected elevated breath hydrogen values after pulse consumption in healthy subjects^(24)^ compared with a diet excluding pulses^(25)^ or to a low-FODMAP control meal^(26)^. Moreover, breath gas measurements have been proposed to have a diagnostic value. For example, lactose and fructose are known to induce higher hydrogen values in individuals with carbohydrate malabsorption^(27,28)^. Yet, whether higher intestinal gas production is related to the perception of gastrointestinal symptoms is currently unclear. Some studies have reported that IBS patients have a higher breath hydrogen response to foods both low and high in FODMAP compounds compared with healthy subjects^(8,29)^, but also results lacking between-volunteer group differences have been presented^(30)^. It is unclear whether the symptoms of IBS patients are linked to visceral hypersensitivity in addition of excessive carbohydrate fermentation^(31)^.

This study investigated, in subjects with a sensitivity towards pulses and controls, whether the *β*-glucan in oat flour would inhibit the fast fermentation of the FODMAP compounds of pulses, and whether the fermentation rate would be linked with perceived gastrointestinal symptoms. In short, a two-leg, 4-day cross-over study on the effects of oat- and rice flours to the fermentation rate of pulses in individuals with a self-reported perception of pulse-induced symptoms and healthy controls by measurements of hydrogen and methane from their breath (4th day) as well as by their gut symptoms and defecation diaries was conducted. Additionally, to further the understanding of consumer mindsets towards pulse products in relation to the resulting gastrointestinal symptoms, to a General Health Interest and to environmental awareness, an online survey was conducted. To our knowledge, this is the first clinical trial investigating the effect of oat fibres, with a known digesta viscosity increasing effect, on the gastrointestinal fermentation rate of pulses as detected from breath hydrogen measurements and self-reported gastrointestinal effects.

## Materials and methods

### Online survey

An online survey was created with the Webropol 3.0 software (Webropol Oy, Helsinki, Finland) in Finnish and English. It was mainly advertised in Turku, Finland, via the University of Turku communication channels but available without restrictions. The respondents (*n* 229) to the survey were encouraged to participate in the clinical trial as well. The survey started with questions evaluating the frequency and reasons to consume different pulses and pulse-containing products, followed by questions evaluating the quality of the possibly perceived symptoms. After these, the survey contained the standardised General Health Interest scale^(32)^ with minor modifications, the Food Neophobia Scale (FNS)^(33)^ and an Environmental Interest Scale, which was created for this study (online Supplementary Table S1).

### Clinical trial

#### Study subjects

The study was designed to include two groups of subjects being those that self-reported to experience gastrointestinal symptoms after consumption of pulses (the sensitive group), and those that self-reported no gastrointestinal problems after consumption of pulses (the control group). The sample size was determined by power calculations based on our earlier study where postprandial excretion of hydrogen was measured as a response to different types of rye bread^(34)^. A sample size of 8 would have 80 % power to detect a difference of 39·5 in breath hydrogen AUC values (very large effect size 1·15) using a paired *t* test with a two-sided significance level of 0·05. Although Pirkola *et al.* did not measure the between volunteer group differences in breath hydrogen, it was calculated that a sample size of 21 would have 80 % power to detect a difference of 36·1 in breath hydrogen AUC values (large effect size 0·80) using an independent *t* test with a one-sided significance level of 0·05. The initially targeted volunteer size for each group was set to 25.

A total of sixty-one subjects were recruited from the area of Turku, Finland, from February 2020 to December 2020 of which fifty were allocated to the interventions (online Supplementary Fig. S1). The clinical trial was terminated in January 2021 when the targeted number of participants had signed the informed consent. Inclusion criteria were self-reported good health, an adult age (18–64) and normal to overweight BMI (18·5–30). Exclusion criteria were allergy to any component of the study products, coeliac disease, use of antibiotics within previous 3 months, use of any medication with gastrointestinal effects (e.g. laxatives or proton pump inhibitors), blood donation or participation in another clinical study within a month, smoking, pregnancy or lactation. Subjects with diagnosed or self-suspected IBS were eligible for the sensitive group. We decided not to test the sensitive group with the Rome Criteria^(35)^ but to include the participants with a subjective experience of pulse-related symptoms for the reasons discussed later. All subjects gave their written informed consent before enrolling in the study. The compatibility of study candidates was assured with interview. Candidates meeting the preliminary inclusion criteria received blood tests (i.e. blood counts, thyroid function tests, total immunoglobulin A and immunoglobulin A antibodies specific to transglutaminase for coeliac diagnostics) and were admitted if the tests were in the normal range. The study protocol was approved by the Ethics Committee of the Hospital District of Southwest Finland. The study was registered in ClinicalTrial.gov (identifier: NCT04273659). Six participants interrupted the trial due to personal reasons, and two were excluded based on the analysis of their food diary (1) or outlying breath results (1). Thus, the study groups consisted of healthy participants (control group, *n* 21) and healthy participants with a subjective experience of gastrointestinal symptoms after consumption of pulses (16) and subjects with IBS (5) and a subjective experience of gastrointestinal symptoms after consumption of pulses (sensitive group, *n* 21). The demographic characteristics of the study groups are presented in Table 1.

**Table 1.** Demographic characters of the subjects of the cross-over trial and online questionnaire

	Cross-over trial				Online survey	
	Sensitive		Control		Respondent	
	Mean	sd	Mean	sd	Mean	sd
Subject (*n*)	21		21		229	
Male/Female	6/15		6/15		34/188*	
Age	28·7	9·5	29·9	9·4	31	12
BMI	23·7	3·2	23·1	2·3	n.a.	
Diet Quality Index	9·7	1·8	9·9	2·3	n.a.	

Age, BMI and Diet Quality index are presented as mean ± sd.

n.a. = not analysed.

* 7 answers of ‘other/I don’t want to tell’.

#### Study products

Two study products were designed being a spoonable, pulse-based product supplemented with either rice flour mixed with rice protein (hereafter rice mix) or fibre-rich oat flour. Two flavours of each were produced with fruit or berry jam. Each participant was given one fruit-flavored and one berry-flavoured product daily. The pulse products were manufactured by Valio Ltd, Helsinki, Finland, for the study purposes only. The flavoured pulse products contained 11·8 % of pea flour (yellow pea, Arolan tila, Finland). The other ingredients were water, jam (Valio Ltd, Suonenjoki), canola oil, Ca, salt and starter culture. Ingredients were mixed and homogenised with an Ultraturrax mixer. The mixture was warmed in a water bath, pasteurised (88°C, 5 min) and cooled to 43°C. A starter culture was added, and the product was fermented with lactic acid bacteria at 43°C, until the pH was under 4·6. The fermented product was cooled to room temperature, and jam was added. The portion size was 125 g containing 14·75 g of pea flour. Products were packaged in white plastic cups, sealed with aluminium foil, frozen and stored at −18°C in identical packages until provided to the study subjects. The flours were mixed in the thawed pulse product before consumption. The flour portions were designed to be matched in energy, fat, proteins and digestible carbohydrates and differed mainly in the amount of dietary fibre. The ingredients for the rice mix and oat flour were purchased commercially.

The nutrient composition of the study products is presented in Table 2. The nutrient compositions of the product with fruit jam, the product with berry jam, rice mix and oat flour were analysed separately. After homogenisation of the sample, the moisture, fat and ash contents were analysed by a gravimetric method according to SFS-EN ISO/IEC 17 025:2017. The protein content was analysed by the Kjeldahl method according to SFS-EN ISO/IEC 17 025:2017. The total carbohydrates and energy content were calculated based on the other nutrient content analyses. The total dietary fibre and the amount of soluble and insoluble fibre were analysed by the enzymatic-gravimetric method according to AOAC 991·43, ISO/IEC 17 025:2005. The amount of verbascose, raffinose and stachyose was analysed with HPAEC-PAD based on ISO-EN 22 184 & IDF 244 and ICUMSA method GS4/8–19 (2005). Glucose, fructose, saccharose, maltose and galactose were analysed with HPAEC-PAD according to ISO/IEC 17 025:2017. The total amount of *β*-glucan was analysed only from oat flour with enzymatic-spectrophotometric method. The analyses were conducted at Eurofins Scientific Finland Ltd (Raisio, Finland).

**Table 2.** Nutrient compositions of the study meals as amount per study product. 7·5 g of rice mix or 9·4 g of oat flour was added to the berry or fruit flavoured pulse product (125 g)

	Pulse product with berry jam + rice mix (132·5 g)	Pulse product with fruit jam + rice mix (132·5 g)	Pulse prodcut with berry jam + oat flour (134·4 g)	Pulse product with fruit jam + oat flour (134·4 g)
Energy (kJ)	529	534	551	556
Energy (Kcal)	125	127	131	132
Moisture (g)	101	102	102	101
Ash (g)	0·8	0·8	1·3	1·3
Protein (g)	5·8	5·7	6·2	6·0
Fat (g)	2·3	2·3	2·7	2·7
Carbohydrates (%)	18·7	19·2	18·2	18·7
of which sugars (%)	7·4	7·8	7·6	8·1
Glucose (g)	1·3	1·0	1·3	1·0
Fructose (g)	1·1	0·05	1·1	0·05
Saccharose (g)	4·9	5·7	5·1	6·0
Lactose (g)	0·05	0·05	0·05	0·05
Maltose (g)	0·07	0·08	0·07	0·08
Galactose (g)	<0·05	<0·05	<0·05	<0·05
Verbascose (%, w/w)	0·4	0·4	0·4	0·4
Raffinose (%, w/w)	<0·3	<0·3	<0·3	<0·3
Stachyose (%, w/w)	0·4	0·4	0·4	0·4
Salt (%)	0·1	0·1	0·1	0·1
Fibre (total) (%)	3·4	3·3	4·5	4·4
Soluble (%, w/w)	0·3	0·3	1·2	1·2
Insoluble (%, w/w)	3·4	3·2	3·6	3·4
Beta-glucan (%, w/w)	n.a	n.a	19·1	1·8

#### Study design

The study was a randomised, double-blind, cross-over, intervention trial. All participants attended two study periods with a ≥ 2 weeks washout period between them. The flow chart of the study is presented as Supplementary Fig. S1. The order of the meal types was randomised by a random number generator. Before each intervention period, the diet was restricted for 2 weeks to not contain any pulses (the run-in period). For the first two days of the intervention period, the diet was restricted to include only food ingredients that cause a low intestinal gas production (a low-FODMAP diet) (online Supplementary Table S2) supplemented with a study product twice a day. For the third day, the low-FODMAP diet was restricted further to be low-phenolic, low-fibre and non-dairy but included the study product twice a day. For the fourth day, after 10 h of fasting, the participants were served breakfast including two portions of the study product with coffee or tea on the University premises. During the fasting state and after the breakfast, the breath hydrogen and methane were measured with a Gastrocheck Gastrolyzer^®^ device (Bedfont, GB) for 8 h at every 15 min. Lunch (rice and chicken or rice and eggs for a vegetarian choice) was served after 4 h of the first postprandial breath gas measurement. The participants were asked to keep a food diary during the first 3 d of the intervention and gut symptom and defecation diaries during the intervention periods (at 4 d).

Each subject received a total of eight study products (four with berry jam and four with fruit jam) during each study period. They were instructed to eat two study products per day, one in the morning and one in the evening during the first 3 d of the study period. On the morning of the fourth day of the intervention, two portions of the study product (one with berry jam, other with fruit jam) were served at once. A research assistant blinded the meals by sealing the flours in plastic bags labelled with numbers 1 or 2.

#### Food diaries

Subjects were given written and oral instructions on filling the food diaries during the 3 first days of each intervention period. Kitchen scales were provided to ensure the accuracy of measurements. The quality of overall diet before the intervention period was assessed by a validated questionnaire for the evaluation of diet quality index^(36)^. The questionnaire contained eighteen questions regarding the frequency and the amount of the consumption of food products during the preceding week. The quality of the diet was defined as poor when index points were less than ten out of the maximum fifteen points and good when points were ten or more.

#### Breath gas measurements

Breath hydrogen and methane were analysed with the Gastrocheck gastrolyzer^®^ device during the fourth day of each intervention. The device was calibrated monthly according to the instructions provided by the manufacturer. The participants exhaled in the mouthpiece of the device every 15 min for 8 h. The time of occurrence and the magnitude of peak gas production, the mean of the five highest gas values and the total area under the gas production curve for each subject were analysed.

#### Gut symptom diary

Gut symptoms diaries were kept during the 4-d interventions. The diary included the type of the symptom (i.e. upper abdominal pain, lower abdominal pain, cramping, bloating, flatulence, rumbling or other type of symptom), the severity of the symptom rated in a scale of 1 to 4 (1: being very mild, 2: mild, 3: moderate and 4: severe) and the duration of the symptom. During the first three days, the diary was divided into time slots of three hours, except during nighttime, which was marked as a six-hour slot (from midnight until 06.00). The fourth day (until the end of the study) was marked in one-hour slots except during the nighttime, which was one six-hour slot. Gut symptoms were evaluated as a sum of the three first intervention days and separately for the fourth day.

#### Defecation diary

Subject filled the defecation diary during the 4-day interventions. The diary was based on the 7-step Bristol scale of stool form^(37)^. Subjects recorded their observations following the Bristol scale and marked the date and time.

### Statistical analysis

#### Online survey

Sum variables were calculated for General Health Interest, FNS and Environmental Interest Scale, and two-step clustering was used to create 2–3 subgroups in each scale. A Pearson’s chi-squared test was used in cross-tabulations of different questions or statements (frequencies) in the subgroups. A Kruskall–Wallis test and a Mann–Whitney’s *U* test were used to examine differences between subgroups in usage questions rated on scales. Statistical analyses were carried out using IBM SPSS Statistics (version 25, USA).

#### Clinical trial

The analyses were done by originally assigned groups (*n* 42). Statistical analyses of the comparisons of the breath gas measurements, gut symptoms and defecations between the study meals and the study groups were carried out using IBM SPSS Statistics 25 software, USA. The comparisons between the groups were made with One-Way ANOVA and with a Mann–Whitney *U* test in case of not normally distributed parameters. The comparisons between the meals were made with a related-samples Wilcoxon signed rank test. The correlations were made with a Spearman correlation test. The effect sizes were calculated with Cohen’s d for normally distributed parameters, with Cliff’s delta or with an effect size calculation for Wilcoxon signed-rank test in case of not normally distributed parameters. The power calculation was executed with G Power, G * Power version 3.1.9.4^(38,39)^. Data are presented as mean +/– sd and as median +/– IDQ in the Supplementary material.

## Results

### Online survey

**Fig. 1.**
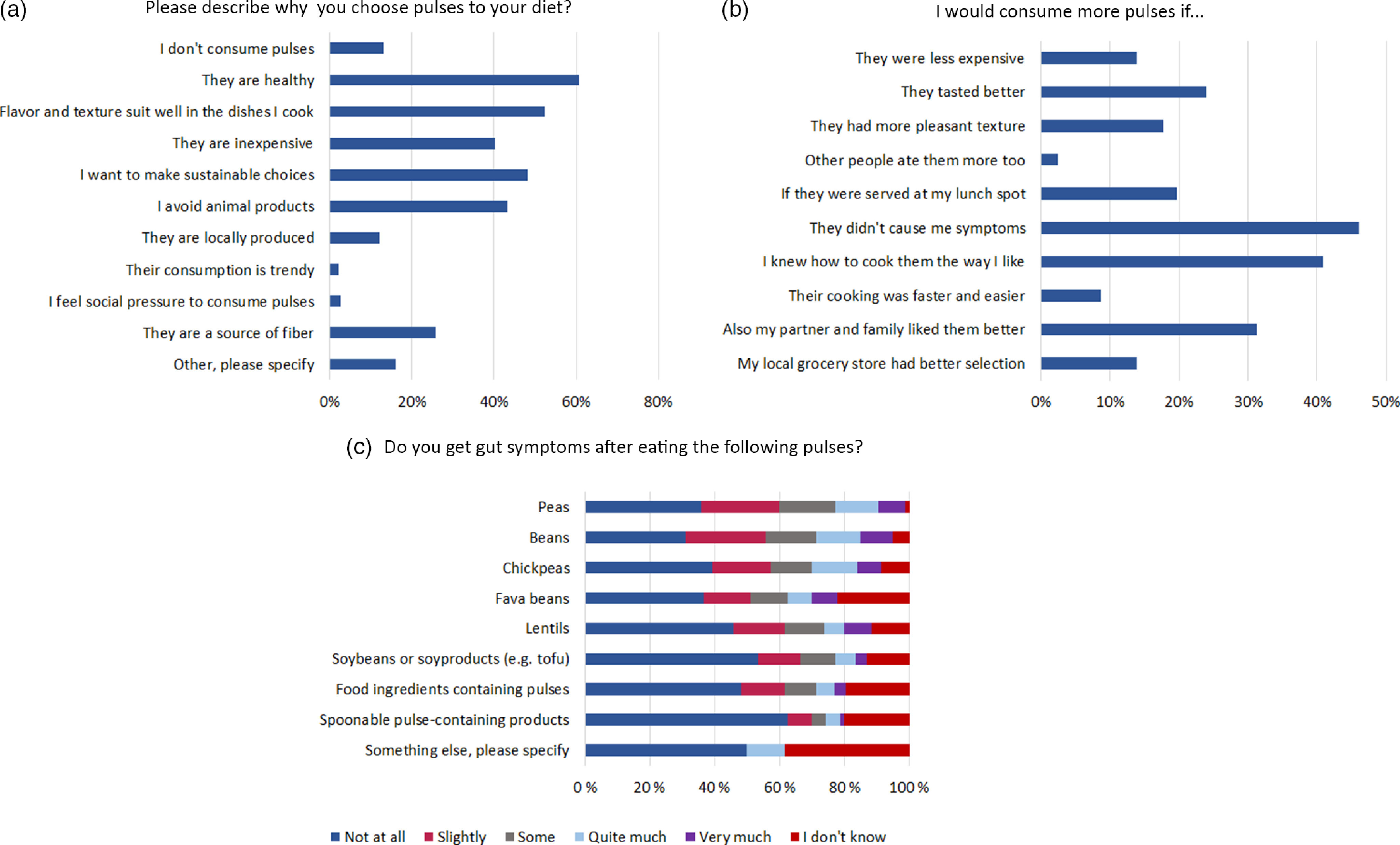
Frequencies of respondent replies to questions/statements from the online survey (*n* 229). Multiple options were possible to choose for questions A and B. One option was chosen for each type of pulse for question C.

Of the respondents (*n* 229), 82 % were women, 40 % were under 25 years old and 63 % were students. The demographic data of the respondents are presented in Table 1. The most common reasons to consume pulses or products made from them (Fig. 1) were their healthiness, chosen by 61 %, the suitability of the flavor and texture to foods (52 %) and the will to make sustainable choices (48 %). The respondents were asked to select ‘What would make them consume more pulses with the most chosen reason being ‘If they would not cause me symptoms,’ chosen by 46 % of respondents (Fig. 1). This was used as an additional grouping for the respondents in addition to grouping based on their answers to scales General Health Interest, FNS and Environmental Interest Scale. Beans, soybeans or soybean-based products and spoonable, pulse-containing products were consumed most often (online Supplementary Table S3). At the same time, peas, beans and lentils were most frequently reported to cause symptoms. Flatulence and bloating were the most reported symptoms (Fig. 1).

Grouping, based on symptoms from pulses (‘Symptoms’ in Table 3) and the general perception on new foods (FNS status), influenced pulse consumption more than interest in health or in the environment (Environmental Interest Scale or General Health Interest status, Table 3). The group that selected symptoms affecting their pulse consumption perceived that their gut symptoms were more severe compared to the group that did not select symptoms affecting their pulse consumption. The symptomatic group selected less often that other reasons than a lack of symptoms could increase their pulse consumption. More food neophobic respondents behaved in the same way as the symptom-perceiving group. It is likely that the will to avoid symptoms was the probable cause of more neophobic behaviour. Symptoms and FNS status had the most impact on the usage of pulses or getting gut symptoms from them (online Supplementary Table S3). The interest in health was also seen in the higher consumption of whole grain cereals, fruits, berries, vegetables and root vegetables (*P* = 0·01).

**Table 3.** Comparison of subgroups formed from participants to the online questionnaire in selected statements and questions

Statement/question	Comparison of subgroups (Pearson’s *χ* ^2^ test; *P*-values and frequencies)							
	Symptoms*		Food neophobia†		General health interest†		Environmental interest†	
	(1 *n* 96; 2 *n* 133)		(1 *n* 94; 2 *n* 89; 3 *n* 46)		(1 *n* 66; 2 *n* 163)		(1 *n* 50; 2 *n* 95; 3 *n* 84)	
Describe why you choose pulses to your diet (Select all that apply; %)								
They are healthy	0·006	50 %; 68 %	0·007	72 %; 55 %; 48 %	0·001	44 %; 68 %	0·007	44 %; 60 %; 71 %
Flavour and texture suit well in the dishes I cook	0·003	41 %; 61 %	0·007	63 %; 51 %; 35 %	ns		ns	
I want to make sustainable choices	0·008	38 %; 56 %	<0·001	63 %; 46 %; 22 %	ns		<0·001	8 %; 40 %; 81 %
I avoid animal products	0·011	33 %; 50 %	ns		ns		<0·001	6 %; 31 %; 80 %
They are inexpensive	ns		0·006	50 %; 39 %; 22 %	ns		<0·001	28 %; 33 %; 56 %
They are a source of fibre	ns		ns		ns		0·029	
Other, specify	ns		ns		ns		ns	
I do not consume pulses	0·044	26 %; 15 %	0·003	14 %; 17 %; 37 %	0·030	29 %; 16 %	<0·001	46 %; 20 %; 4 %
They are locally produced	ns		ns		ns		<0·001	0 %; 10 %; 23 %
I feel social pressure to consume pulses	ns		ns		ns		ns	
Their consumption is trendy	ns		ns		ns		ns	
*I would consume more pulses (Select all that apply; %)* ‡	(1 *n* 96; 2 *n* 112)		(1 *n* 84; 2 *n* 79; 3 *n* 45)		(1 *n* 58; 2 *n* 150)		(1 *n* 48; 2 *n* 89; 3 *n* 71)	
If they did not cause me symptoms*	<0·001	100 %; 0 %	<0·001	33 %; 44 %; 73 %	ns		ns	
If I knew how to cook them the way I like	<0·001	27 %; 53 %	ns		ns		ns	
If my partner/family liked them better	<0·001	15 %; 46 %	0·045	39 %; 30 %; 18 %	ns		ns	
If they tasted better	ns		ns		ns		ns	
If their availability in the cafeteria of my workplace was better	0·008	12 %; 27 %	0·031	26 %; 20 %; 7 %	ns		0·002	8 %; 16 %; 32 %
If they had more pleasant texture	ns		ns		ns		ns	
If my local grocery store had better selection	0·043	8 %; 19 %	ns		ns		ns	
If they were less expensive	0·015	7 %; 20 %	ns		ns		ns	
If their cooking was faster and easier	ns		ns		ns		ns	
If other people ate them more too	ns		ns		ns		ns	
How would you describe the symptoms caused by the pulses and their intensity? (Rate intensity scale: 1 – not at all, 2 – mild, 3 – moderate, 4 – severe, 5 – very severe)								
	(1 *n* 96; 2 *n* 133)		(1 *n* 94; 2 *n* 89; 3 *n* 46)		(1 *n* 66; 2 *n* 163)		(1 *n* 50; 2 *n* 95; 3 *n* 84)	
Upper gut pain	<0·001§		0·027||		0·046||		0·004||	
Lower gut pain	<0·001§		0·004||		ns		ns	
Cramps	<0·001§		0·008||		ns		ns	
Bloating	<0·001§		<0·001||		ns		ns	
Flatulence	<0·001§		ns		ns		ns	
Rumbling	<0·001§		ns		ns		ns	
Diarrhoea	<0·001§		ns		ns		ns	
Constipation	<0·001§		0·002||		ns		ns	
Nausea	<0·001§		ns		ns		ns	

* Grouping based on answers on the statement ‘if they didn’t cause me symptoms’; group 1 = statement selected; group 2 = not selected.

† Statements included in the scales are in the Supporting Table S2. Grouping based on the two-step cluster analysis of the scale sum variables. Group 1: low food neophobia/health interest/environmental interest; group 3 (or 2 in GHI): high neophobia/health interest/environmental interest. Frequencies (%) shown after *P*-values if a significance was observed; ns: *P*-value above 0.05.

‡ Twenty-one participants did not select any statements to consume more pulses.

§ Direction of the statistical significance: group 1 gave higher ratings than group 2.

|| Direction of the statistical significance: high food neophobia (group 3) or health interest (2) gave higher ratings. The lower environmental interest group (1) gave higher ratings.

### Clinical trial

#### Diet of the study subjects

The overall baseline diet assessed by the validated index of diet quality questionnaire was considered good in most of the study subjects, and the average was close to 10 in both groups (Table 1). Based on the food diaries, the participants followed the dietary instructions carefully. One participant was excluded based on the food diary data for the reason of not following the FODMAP-free diet during the instructed period. The vegetarian option for lunch, being rice and eggs, was chosen by 5 sensitive subjects and 2 control subjects. The lunch choice did not affect breath hydrogen or methane AUC values of the subjects. The breath hydrogen AUC mean ± sd of subjects who chose the egg option was 37·9 ± 10·7, and from those who chose the chicken option, the AUC mean ± sd was 37·1 ± 25·1 after consumption of the rice mix meal (*P* = 0·938) and 84·8 ± 23·2 (egg) and 83·8 ± 40·3 (chicken) after consumption of the oat flour meal (*P* = 0·956). Breath methane AUC values mean ± sd of subjects who chose the egg option were 13·4 ± 26·2, and from those that chose the chicken option, the AUC mean ± sd was 44·6 ± 74·5 after consumption of the rice mix meal (*P* = 0·319) and 17·1 ± 34·4 (egg) and 44·5 ± 75·9 (chicken) after consumption of the oat flour meal (*P* = 0·394). Comparisons were done with One-Way ANOVA.

#### Breath gas measurements

**Fig. 2.**
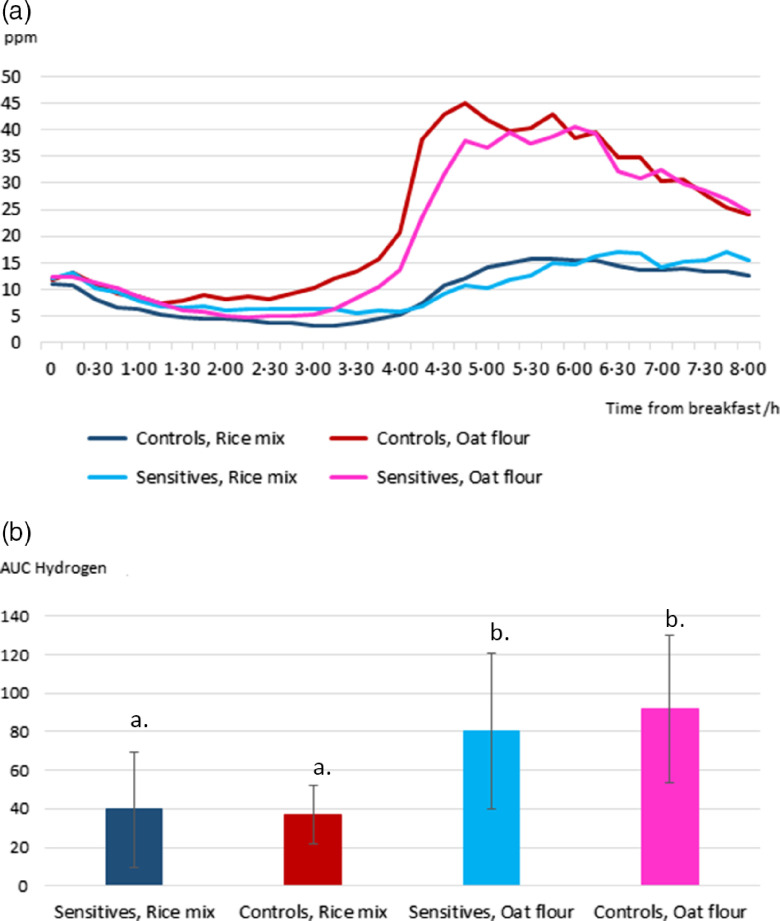
(a) The average hydrogen production rate during the fourth study day (*n* 42). Lunch was served at the time point of 4 h. (b) Area under curve values of hydrogen production (mean +/– sd). a differs significantly from b (*P* < 0·05) (Wilcoxon Rank Test). The data are presented as median + IQR in Supplementary Fig. S3 and in Supplementary Table S4.

The mean values ± sd of breath gas measurements are presented in Table 4A (Medians and Interquartile ranges Supplementary Table 4S). The AUC of hydrogen production was higher after the meal with oat flour compared with the meal with rice mix in both the sensitive and control groups (*P* = 0·001 and *P* = 0·001, respectively, Wilcoxon rank test, Wilcoxon rank effect sizes 0·59 and 0·58, respectively, medium size effect on Cohen’s scale) (Fig. 2, Table 4A, online Supplementary Fig. S3 and Table S4). The same applied for the highest value of the hydrogen production (*P* = 0·001 and *P* = 0·001, respectively, to the groups) and the average of five highest values (*P* = 0·001 and *P* = 0·001, respectively, to the groups). The time of the hydrogen peak was on average between 5 and 6 h after the zero point and did not differ between the groups after the rice mix meal (*P* = 0·533) or after the oat flour meal (*P* = 0·123) or between the meals in the sensitive group (*P* = 0·370) or in the control group (*P* = 0·067). No difference was observed in the AUC values of hydrogen production between the sensitive and the control groups after the rice mix or oat flour meals (*P* = 0·483, *P* = 0·541, respectively, One-Way ANOVA, Cohen’s d = 0·18 and 0·28, respectively, small effect). The AUC of methane production did not differ between the meals in the sensitive group (*P* = 0·058) nor in the control group (*P* = 0·737) (Wilcoxon Rank test). There was no difference in the highest value of methane production in the sensitive or in the control groups (*P* = 0·657, *P* = 0·963, respectively) or in the average of 5 highest values of methane production (*P* = 0·499, *P* = 1·000, respectively). The AUC values of methane production did not differ between the sensitive and the control groups after the rice mix or the oat flour meals (*P* = 0·759, *P* = 0·473, respectively, One-Way ANOVA). Eight sensitive and five control subjects were considered as methane producers since their methane response of a single measurement was greater than or equal to 2 ppm. When only methane producers were compared, the AUC values did not differ between the sensitive and the control groups after the rice mix or the oat flour meals (*P* = 0·446, *P* = 0·935, respectively, One-Way ANOVA) or between the meals in the sensitive group (*P* = 0·327) or in the control group (*P* = 0·225) (Wilcoxon Rank test). No clear peaking was observed in the methane production, and thus, the peaking time was not compared.

**Table 4.** (A) Breath gas measurements; (B) gut symptoms and (C) defecation pattern by the Bristol stool scale during the study period (Mean values and standard deviations). The data are presented as median + IQR in Supplementary Table S4

	Sensitives, Rice Mix		Sensitives, Oat flour		Controls, Rice mix		Controls, Oat flour	
	Mean	sd	Mean	sd	Mean	sd	Mean	sd
A. Breath gas measurements								
H2 AUC	39·8	30·0	80·3	40·7	37·0	15·4	91·7	38·2
H2 highest value	27·7	14·5	61·5	26·8	25·8	11·9	69·1	32·6
H2 mean of 5 highest values	22·1	12·5	51·8	25·3	21·4	10·0	59·6	23·7
H2 time of peak value (h)	6·2	1·9	5·7	1·1	5·4	2·5	5·2	0·9
CH4 AUC	43·5	70·9	48·6	80·9	37·4	73·5	32·9	63·5
CH4 highest value	17·6	25·3	18·9	33·5	13·0	24·7	12·2	22·2
CH4 mean of 5 highest values	15·2	23·3	16·6	28·4	11·9	22·2	11·3	20·8
CH4 time of peak value (h)	n.a.		n.a.		n.a.		n.a.	
B. Gut symptoms								
Sum of symptoms of 3 first days	15·9	15·3	15·8	16·7	2·1	0	2·5	3·5
Sum of symptoms of the 4th day	5·7	6·3	8·2	7·2	0·3	0·7	1·3	1·8
Very mild symptoms, 3 first days	3·9	4·2	4·4	5·3	1·4	2·8	1·7	2·6
Mild symptoms, 3 first days	4·8	5·6	5·4	6·6	0·5	2·3	1·0	1·9
Moderate symptoms, 3 first days	3·3	4·3	4·8	7·0	0·2	0·9	0·4	1·0
Severe symptoms, 3 first days	3·7	8·5	1	2·6	0·04	0·6	0·1	0·4
Very mild symptoms, 4th day	1·9	2·5	2·9	3·2	0·3	0·6	0·8	1·3
Mild symptoms, 4th day	1·9	2·5	2·9	3·2	0·3	0·6	0·4	0·9
Moderate symptoms, 4th day	1·7	3·2	1·5	2·7	0		0·1	0·4
Severe symptoms, 4th day	0·2	0·4	0·6	1·2	0		0	
Upper abdomen pain, 3 first days	1·1	2·0	1·3	2·3	0·2	0·6	0	
Lower abdomen pain, 3 first days	2·0	3·5	1·6	3·5	0·2	0·6	0·3	0·9
Cramping, 3 first days	0·4	1·0	0·6	1·5	0		0·05	0·2
Bloating, 3 first days	4·4	5·4	4·2	5·7	0·6	1·2	0·6	1·1
Flatulence, 3 first days	3·7	3·4	4·1	4·4	0·7	1·3	1·3	1·9
Rumbling, 3 first days	2·1	2·0	1·9	2·2	0·2	0·5	0·5	1·1
Other symptoms, 3 first days	2·3	6·1	2·0	5·5	0·2	0·9	0	
Upper abdomen pain, 4th day	0·5	0·7	0·7	1·7	0		0	
Lower abdomen pain, 4th day	0·9	1·7	0·8	1·9	0·04	0·2	0·1	0·5
Cramping, 4th day	0·1	0·4	0·2	0·5	0		0·05	0·2
Bloating, 4th day	1·8	3·1	2·5	3·4	0·05	0·2	0·2	0·5
Flatulence, 4th day	1·0	1·2	2·3	2·2	0·1	0·3	0·4	0·6
Rumbling, 4th day	0·5	1·1	0·9	1·8	0·1	0·3	0·3	0·7
Other symptoms, 3 first days	0·9	3·0	0·9	1·3	0·05	0·2	0·1	0·4
C. Defecation pattern by Bristol stool scale during the study period								
1	0·9	1·7	1·0	1·7	0·4	0·8	0·1	0·3
2	0·2	0·4	0·4	0·9	0·2	0·5	0·2	0·7
3	0·3	0·7	0·6	1·0	0·6	0·7	1·0	1·2
4	1·0	1·1	0·8	1·0	1·4	1·3	1·9	1·6
5	2·6	2·3	2·5	2·4	1·3	1·7	1·3	2·0
6	0·6	0·9	0·6	1·0	0·2	0·5	0·3	0·6
7	0·2	0·4	0·3	0·8	0		0·05	0·2
Total	5·8	3·2	6·3	3·5	3·9	1·9	4·7	2·1

H2 = hydrogen; AUC = area under curve; CH_4_ = methane; n.a. = not analysed. No clear peaking point could be determined for methane values.

#### Gut symptom and defecation diaries

**Fig. 3.**
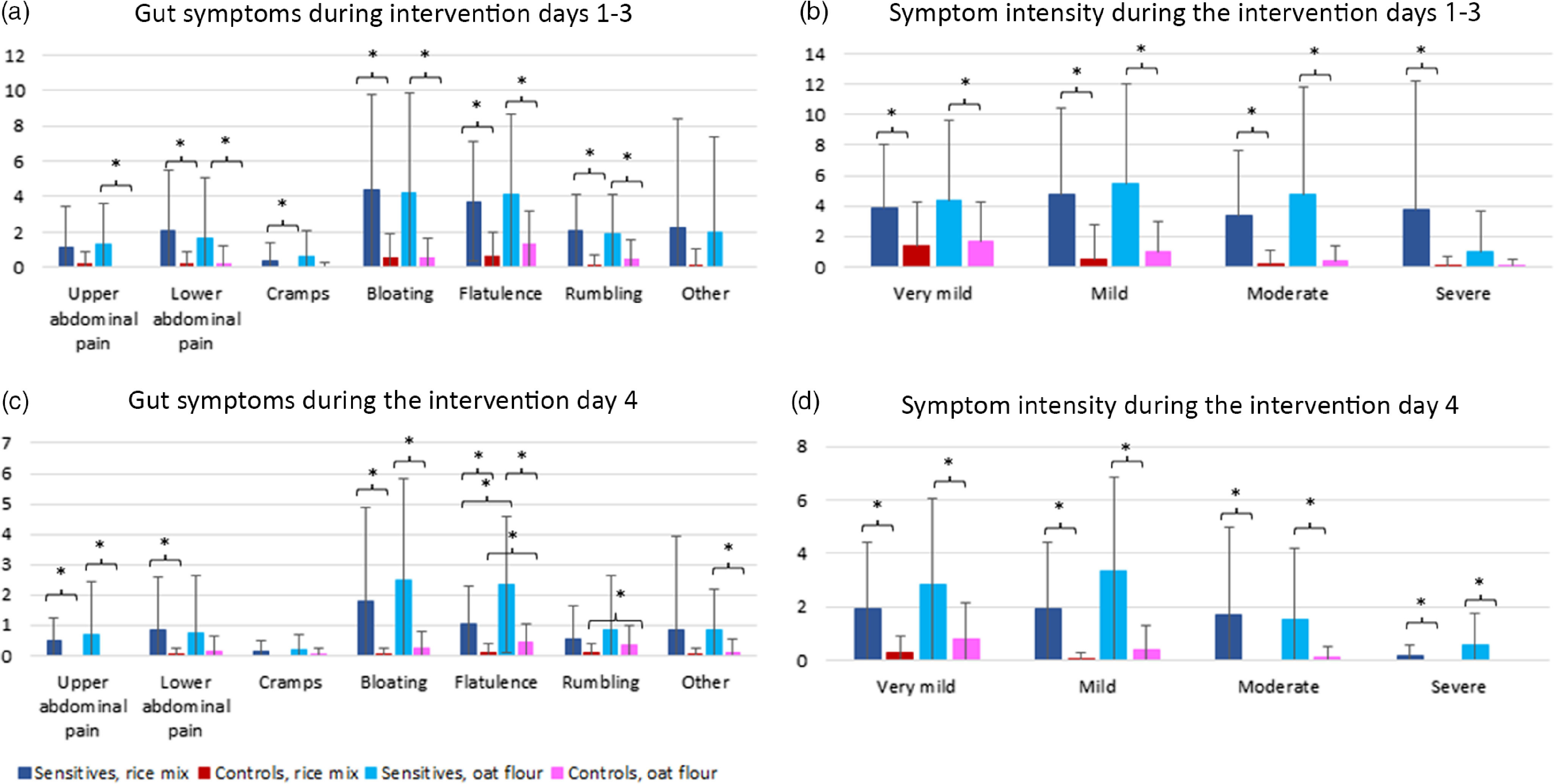
(a) The average number of reported gut symptoms during the intervention days 1–3. (b) The average number of reported symptom intensities during the intervention days 1–3. (c) The average number of reported gut symptoms during the intervention day 4. (d) The average number of reported symptom intensities during the intervention day 4. Values are mean of 21 ± sd. Significant differences (*P* < 0·05) are marked with × (Mann–Whitney *U* test, Wilcoxon Rank test). The data are presented as median + IQR in Supplementary Table S4.

The mean values ± sd of gut symptoms are presented in Table 4B and medians and interquartile ranges in Supplementary Table 4S. The sensitive group reported more symptoms in total compared with the control group during the first three days of study period (*P* = 0·001, *P* = 0·001, respectively; Mann–Whitney *U* test, Cliff’d delta = –0·76 and –0·68, respectively, medium effect) and during the fourth day (*P* = 0·001, *P* = 0·001, respectively; Mann–Whitney *U* test, Cliff’s delta = –0·67 and –0·68, respectively, medium effect) after both the rice mix and the oat flour meals (Fig. 3, Table 4B, online Supplementary Table 4S). The sensitive group also reported more symptoms of each state of intensity, being very mild, mild, moderate and severe, compared with the control group after both the meal with rice mix (during the first 3 study days: *P* = 0·01, *P* = 0·001, *P* = 0·001, *P* = 0·008, respectively, to intensity; and during the fourth day: *P* = 0·001, *P* = 0·001, *P* = 0·001, *P* = 0·038, respectively, to intensity) and the meal with oat flour (during the first 3 study days: *P* = 0·038, *P* = 0·001, *P* = 0·001, *P* = 0·140 (not significant), respectively, to intensity; and during the fourth day: *P* = 0·008, *P* = 0·001, *P* = 0·002, *P* = 0·019, respectively, to intensity), (Mann–Whitney *U* test). The sensitive group also reported each type of symptom, being upper abdomen pain, lower abdomen pain, cramping, bloating, flatulence, rumbling and other symptoms more frequent than the control group after both the meal with rice mix (during the first 3 study days: *P* = 0·213 (not significant), *P* = 0·015, *P* = 0·038, *P* = 0·002, *P* = 0·001, *P* = 0·001, *P* = 0·079 (not significant), respectively, to the symptom, and during the fourth day: *P* = 0·004, *P* = 0·035, *P* = 0·076 (not significant), *P* = 0·003, *P* = 0·002, *P* = 0·301 (not significant), *P* = 0·146 (not significant), respectively, to the symptom) and the meal with oat flour (during the first 3 study days: *P* = 0·039, *P* = 0·044, *P* = 0·307 (no significant), *P* = 0·023, *P* = 0·020, *P* = 0·015, *P* = 0·124 (not significant), respectively, to the symptom, and during the fourth day *P* = 0·038, *P* = 0·123 (not significant), *P* = 0·288 (not significant), *P* = 0·010, *P* = 0·001, *P* = 0·536 (not significant), *P* = 0·018, respectively, to the symptom), (Mann–Whitney *U* test) (Fig. 3, online Supplementary Table S4). Nausea and heartburn were most frequently reported as other symptoms. Overall, controls reported very few symptoms.

The perceived frequency, intensity and quality of the symptoms did not differ among the meals during the first three study days in either group (Wilcoxon Rank test) (Fig. 3(a) and (b), Table 4B, online Supplementary Table S4).

Both the sensitive and the control groups reported more flatulence during the fourth study day after the oat flour meal compared with the rice mix meal (*P* = 0·006, *P* = 0·008, respectively, Wilcoxon Rank test; Fig. 3(c); Table 4B, online Supplementary Table S4). The control group also reported more rumbling after the oat flour meal compared with the rice mix meal (*P* = 0·025; Fig. 3(c), Table 4B, online Supplementary Table S4).

Both groups also reported more very mild or mild symptoms during the fourth study day after the oat flour meal compared with rice mix meal (sensitive group: very mild symptoms, *P* = 0·006; mild symptoms, *P* = 0·001; control group: very mild symptoms (1), *P* = 0·027; mild symptoms, *P* = 0·034, Wilcoxon rank test Fig. 3(d), Table 4B, Supplementary Table S4). Overall, no difference was seen in total amount of moderate (intensity (3)) or severe (intensity (4)) symptoms during the fourth study day between the oat and rice mix meals (sensitive group: *P* = 0·754; *P* = 0·071, respectively; control group: *P* = 0·317, *P* = 1·000, respectively; Fig. 3(d), Table 4B, online Supplementary Table S4) indicating that the increased flatulence and rumbling were mainly mild.

A positive correlation was observed between the perceived flatulence and the AUC of hydrogen production after the oat flour meal in both the sensitive group and the control group (*P* = 0·042, *P* = 0·003, respectively; Spearman’s *r* = 0·447, *r* = 0·616, respectively). After the rice mix meal, there was no such correlation (*P* = 0·746, *P* = 0·560, respectively; Spearman’s *r* = 0·075, *r* = 0·135, respectively).

**Fig. 4.**
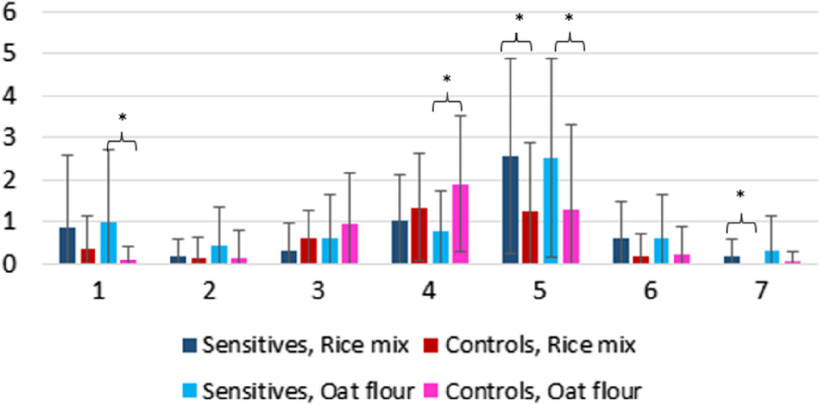
The number of stools during the 4-d intervention period by the Bristol scale. Values are mean of 21 ± sd. Significant differences (*P* < 0·05) are marked with × (Mann–Whitney *U* test). The data are presented as median + IQR in Supplementary Table S4.

The mean values ± sd of defecation patterns are presented in Table 4C (Median and interquartile ranges as online Supplementary Table 4S). The sensitive group reported more defecations at both extremity ends of the Bristol scale compared with the control group during both study periods (Fig. 4, Table 4C, online Supplementary Table S4). After the rice mix meal, the sensitive group reported a statistically higher number of type 5 and type 7 stools compared with the controls (*P* = 0·045; *P* = 0·042, respectively). After the oat flour meal, the control group reported more of type 4 stools compared with the sensitive group (*P* = 0·011), and the sensitive group reported more of type 1 and type 5 stools compared with the controls (*P* = 0·052, *P* = 0·019, respectively). After the rice mix meal, the sensitive group tended to report a more frequent stool pattern compared with controls (*P* = 0·064), but after the oat flour meal, there was no difference (*P* = 0·207). The defecations did not differ between the meals in either the sensitive or the control groups. The defecation patterns between the groups were compared with Mann–Whitney *U* test.

## Discussion

### Online survey

Respondents of the online survey perceived the use of pulses as healthy, inexpensive and sustainable, which was in line with other recent consumer studies^(40,41)^ assessed consumer mindsets towards alternatives of meat and found that legumes, such as chickpeas, beans, peas and lentils, were considered the most natural, appetizing, healthy, edible, ethical, sustainable, least processed and overall, the most positive and familiar products compared with other meat alternatives, such as seitan, insects and lab-grown meat. Smiglak-Krajewska *et al.* (2020) concluded, in their questionnaire study, that peas, beans, soybeans and lentils were the most recognised and most frequently consumed legumes. In their study, the most important motives to purchase legumes were flavour, price, protein and fibre contents, and the fact that these products can be an alternative to meat products. Neither of these surveys included questions on the gastrointestinal problems related to the consumption of pulses. According to the results of our survey, pulse-related symptoms are not uncommon among consumers. Close to half of our respondents (46 %) would consume more pulses if they did not cause them symptoms. More processed pulse products, such as spoonable products as well as tofu and other soya products, were tolerated most. It must be noted that participation in the survey was based on interest and likely to attract persons who experienced problems with pulse consumption. As the survey was not randomised and offered to the whole adult population, the results cannot be generalised to the whole Finnish population. Still, the results clearly indicate that the perceived gut symptoms do prohibit the pulse consumption in a group of consumers, and there is interest for pulse products suitable for a sensitive gut.

### Breath hydrogen

The pulse meal with oat flour induced higher hydrogen production compared to the meal with the rice mix. Since the meals mainly differed in the amount of soluble fibre, the increased hydrogen production resulted from the fermentation of the fibre. Others have reported a positive correlation between AUC of hydrogen production and the amount of ingested dietary fibre from rye in healthy adults^(42–44)^. It has also been suggested that breath hydrogen may be linked to the short-chain fatty acid (SCFA) production, since a higher concentration of SCFA and elevated breath hydrogen were measured from both plasma^(42)^ and feces^(43)^ after consumption of soluble fibre. Although SCFA were not measured in the current study, the consumption of oats has been previously reported to increase the production of SCFA^(19)^. Despite earlier studies on the associations between fibre-rich meals and increased production of hydrogen, the present study is the first one observing that the intestinal gas production resulting from soluble fibre does not differ between the subjects with gut sensitivity and controls.

### Gut symptoms and their relation to breath hydrogen

It was hypothesised that an increased viscosity of digesta induced by oat fibres could slow down the fermentation of pulses and thus reduce the symptoms of sensitive subjects. However, we observed no difference in the gas production between the sensitive and the control groups despite the fact that the sensitive group perceived more frequent and intense symptoms compared with the controls after both study meals. As the two meals did not cause a difference in moderate or severe symptoms, both flours were well tolerated, and the symptoms of the sensitive group were induced by the pulse-containing part of the study meals known to contain FODMAP compounds. Moreover, the gut symptoms and the defecation pattern of the sensitive group resembled that of IBS patients^(35)^. Earlier, two different hypotheses have been presented to explain the symptoms resulting from FODMAP compounds in IBS patients. The large bowel hypothesis suggests that as the FODMAP are poorly digested in the small intestine, they drift to the colon and are fermented by the colon microbes. The rapid fermentation produces gas, which induces the intestinal symptoms, such as bloating, flatulence and pain^(30)^. Studies supporting the large bowel hypothesis have reported higher hydrogen production by IBS patients compared with healthy subjects after consumption of both FODMAP-rich and FODMAP-poor foods^(8,29)^. We did not detect more hydrogen in the sensitive group compared with controls, but increased hydrogen production correlated with increased perception of flatulence after the oat flour meal in both the sensitive and control groups. This indicates that colonic fermentation plays a role in the symptom triggering but is not solely causing it.

The small bowel hypothesis claims that the FODMAP are osmotically active and thus draw water to the small intestine. High intestinal water content induces bloating and discomfort, accelerates the orocaecal transit and reduces the absorption from the small intestine^(30)^. We observed no difference in the gas production between the sensitive and the control subjects, which implies that the symptoms of the sensitive group cannot be explained only by more intense colonic fermentation, but by visceral hypersensitivity, i.e. the sensitivity to intestinal distension induced by gas or water. In addition, the small bowel hypothesis offers an explanation about why oats were well tolerated among the sensitive individuals despite increased colonic fermentation. In the small bowel, oat fibres, mainly consisting of soluble *β*-glucan with long polymer chains, increase the viscosity of the digesta instead of drawing water to the intestine^(45)^. Along with the increase of the viscosity of the digesta, the water trapped in the colon will soften the colonic contents and reduce colonic distension^(46)^. Furthermore, the long chains of *β*-glucan are less readily fermented than FODMAP compounds, such as fructo-oligosaccharides and galacto-oligosaccharides with small molecule structures. The rate of fermentation plays a key role in the tolerance of oats, since the gas is produced over a longer time period and the colonic extension is less sudden.

### The hydrogen peak value and small intestinal bacterial overgrowth

There has been a discussion whether IBS could be linked to SIBO, as a greater gas production in the small intestine would cause IBS types of symptoms^(47)^. In the present study, the time of the gas peak did not differ between the sensitive subjects and the controls, thus indicating that the fermentation did not start earlier in the gastrointestinal tract in the sensitive group. However, it must be noted that the time of stomach emptying and further digestion varies among individuals and cannot be measured without ingested probes^(48,49)^. Alterations of the microbiota of IBS patients have also been considered in the discussions of IBS and SIBO^(21,22)^. Yet, since the gut microbiota is known to be influenced by the type and amount of carbohydrates consumed^(50)^, it cannot be ruled out that the changes were rather caused by the low-FODMAP diet than IBS. Indeed, the observed microbiota alterations of the IBS patients in the earlier studies are similar to those observed in coeliac disease patients and healthy subjects following a gluten-free diet, which similarly excludes wheat, rye and barley. An increase of the abundance of *Bacteroides* and a decrease in the abundance of *Bifidobacteria* are observed in both IBS- and coeliac disease patients who avoid the common sources of whole grain^(21,22,51,52)^. Conversely, no such alterations were observed in the microbiota of coeliac disease patients and in individuals with a non-coeliac gluten sensitivity following a gluten-free diet including oats^(20)^. Additionally, FODMAP, such as fructo-oligosaccharides and galacto-oligosaccharides, are known to stimulate the growth of beneficial microbes^(53)^.

### The selection of study subjects

We included subjects to the ‘sensitive’ group based on their subjective experience of pulse-related symptoms instead of a certain medical diagnosis. IBS does not have a physiological marker, yet the diagnosis is based on the exclusion of other diseases and by the patient fulfilling the Rome criteria^(35)^. The first consensus of the IBS criteria was first reached in 1989 and was based on four symptoms frequent in IBS patients being bloating, pain relief with bowel action and more frequent and looser stools with the onset of pain. Later, the criteria have been re-defined several times, and the Rome I, II, III and IV criteria have minor differences in the spectrum and frequency of the symptoms^(35)^. Since our aim for the study was not to investigate whether the sensitive subjects would fulfill the Rome IV criteria, but to observe the responses of self-reported sensitive subjects to the pulse products, we chose to include subjects by their subjective experience of pulse-related symptoms and by the exclusion of coeliac disease by blood screening.

### Conclusions

To conclude, according to the online survey, pulse-related gastrointestinal problems are common, and the more processed pulse products were, as reported, better tolerated. Thus, there is a need for novel processing approaches to ease the digestion of pulse products. Simultaneous ingestion of oat flour, together with pulses, did not reduce but neither worsen the pulse-related symptoms in the sensitive subjects during the first three study days. Although more very mild to mild symptoms were detected on day 4 after the oat + pulses meal compared with pulses + rice flour meal, oats may be a well-tolerated full-grain- and fibre source for also those with a sensitive gut despite increased colonic fermentation. Furthermore, the colonic fermentation of oats is likely to be beneficial especially among the individuals following a fibre-intake restricting, low-FODMAP diet. In this study, gastrointestinal symptoms of subjects sensitive to pulses were not explained by breath hydrogen levels, although a correlation between breath hydrogen and increased flatulence was seen. This suggests that both visceral sensitivity and colonic fermentation play a role in the symptom perception. The link between intestinal hydrogen and SCFA production in the fermentation of the oat-soluble fibre should be studied further. Future studies are also required to examine the symptom-triggering mechanism of FODMAP in the IBS patients.

## Supplementary material

For supplementary material accompanying this paper visit https://doi.org/10.1017/S0007114522000332. click here to view supplementary material
