# Supplementary material for: Effect of oat or rice flour on pulse-induced gastrointestinal symptoms and breath hydrogen in subjects sensitive to pulses and controls – a randomised cross-over trial with two parallel groups
Source: Br J Nutr. 2022 Jan 28;128(11):2181–92. doi: 10.1017/S0007114522000332 (PMC9661369; doi:10.1017/S0007114522000332)
Supplement: Supplementary file 1 [file S0007114522000332sup.zip › S0007114522000319sup002.docx]

**Supplementary Information 1**

Data values were first aggregated to mean values for each EA, and mean coordinates were computed for each EA (EA centroid). One single value was a probable outlier (3 x IQR) according to the criteria of Tukey (Tukey 1977) ^(1)^. This was removed from the data for variogram estimation, but was returned for spatial prediction by kriging. This is because estimates of the variogram are particularly susceptible to extreme values ^(2)^.

Because of the large extent of the sampled region, all spatial analyses were done using the latitude and longitude recorded for the observations, rather than on a rectilinear grid since no single projection would be suitable at all locations. Under the usual assumptions of stationarity in geostatistics, spatial dependence is modelled in terms of the vector that separates locations, or lag. In this setting the lag distance between any two points was computed as the great circle distance on a spherical approximation. This was computed using the *distVincenty Sphere* function from the geosphere package for the R platform ^(3)^. The function *final Bearing* from the same package was used to obtain bearings. Note that, when distances between locations are measured on the sphere, then not all variogram functions that are suitable for use on the plane can be applied. On the sphere the exponential model is authorized ^(4)^, and so it was used for all variogram modelling in this study.

The exponential variogram model describes spatial dependence, taking values which increase with distance. The shape of the model is described by a distance parameter, φ, at distances larger than about 3×φ, the variogram increases only slightly. This distance is called the range of spatial dependence, points separated by longer distances are, in effect, independent.

In addition, variogram values were estimated using the robust estimators due to Dowd (1984) ^(5)^ and to Cressie and Hawkins (1980) ^(6)^. These alternative estimators were considered because, even if marginal outliers (unusual data as they appear on the overall distribution) were removed, spatial outliers (observations unusual in their spatial context, but not necessarily on the overall distribution) can inflate estimates of the variance ^(2)^. An exponential variogram model ^(7)^ was fitted by weighted least squares.

In the absence of spatial dependence, the variogram is a constant (known in geostatistics as a `pure nugget’ variogram); for a spatially-dependent variable the variogram increases with the lag distance. The strength of evidence for spatial dependence in each variable was assessed by fitting a pure nugget model by the same weighted least-squares criterion used to fit the exponential. While the weighted sum of squares for the more complex exponential model is necessarily no larger than that for the pure nugget model, the assessment of whether the difference in fit is evidence for spatial dependence is based on Akaike’s information criterion (AIC). This was computed following Webster and McBratney (1989) ^(8)^. The AIC is based on the weighted sum of squares of the model fit, with a penalty for complexity. When comparing two models one selects that which has the smallest value of AIC.

If the fitted exponential variograms for the variables under study here had smaller AIC than the alternative pure nugget models then they were tested by cross-validation, which was also used to select between the models fitted to output from different estimators. Cross-validation is a standard statistical technique and is used to test the goodness of fit of the variogram model that has been used. Cross-validation is applied by removing each datum from the data set, one at a time, and estimating its value based on the surrounding data, by OK.

The squared cross-validation error at each site was standardized by dividing the ordinary kriging variance. The median standardized squared prediction error (SSPE) has an expected value of 0.455, and a 95% confidence interval [0.337,0.573] (n=1290), in the case of a valid underlying variogram model with kriging errors that appear normally distributed ^(1)^ . The estimator due to Matheron (1962) ^(9)^ is more statistically efficient than the robust alternatives, so if the model fitted to these estimates appeared correct from the cross-validation results (median SSPE) then the alternatives were not considered. If the SSPE suggests that the model fitted to Matheron estimates are affected by outliers, then the models fitted to robust estimates are also cross-validated, and one is selected on the cross-validation results. This procedure follows the recommendations of Lark (2000) ^(2)^. If an exponential variogram (rather than pure nugget) was selected for a variable, this is evidence for its spatial dependence. The selected model, from among those fitted to outputs from different estimators, was then used to compute OK predictions at nodes on a 60-m square grid.

References

1. Tukey JW (1977) Exploratory Data Analysis. Addison-Wesley: Reading, MA, USA.

2. Lark RM (2000) A comparison of some robust estimators of the variogram for use in soil survey. *Eur J Soil Sci* **51**, 137 – 157.

3. Hijmans RJ (2017) Geosphere: Spherical Trigonometry. R Package Version 1.5-7. Available online: https: //CRAN.R-project.org/package=geosphere.

4. Gneiting T (2013) Strictly and non-strictly positive definite functions on spheres. *Bernoulli* **19**, 1327–1349.

5. Dowd PA (1984) The variogram and kriging: Robust and resistant estimators. In Geostatistics for Natural Resources Characterization; Verly, G., David, M., Journel, A.G., Marechal, A., Eds.; Reidel: Dordrecht, the Netherlands, pp. 91–106.

6. Cressie N & Hawkins DM (1980) Robust estimation of the variogram. *Math Geol* **12**, 115–125.

7. Oliver MA & Webster R (2015) Basic Steps in Geostatistics: The Variogram and Kriging; Springer: Cham, Switzerland.

8. Webster R & McBratney AB (1989) On the Akaike Information Criterion for choosing models for variograms of soil properties. *J Soil Sci* **40**, 493–6.

9. Matheron G (1962) Traité de Géostatistique Appliqué, Tome 1. Memoires du Bureau de Recherches Géologiques et Minières; Technip: Paris, France.
