# Supplementary figures and images for: Effect of oat or rice flour on pulse-induced gastrointestinal symptoms and breath hydrogen in subjects sensitive to pulses and controls – a randomised cross-over trial with two parallel groups
Source: Br J Nutr. 2022 Jan 28;128(11):2181–92. doi: 10.1017/S0007114522000332 (PMC9661369; doi:10.1017/S0007114522000332)

**Supplementary Figure 1**. Summary statistics for WRA


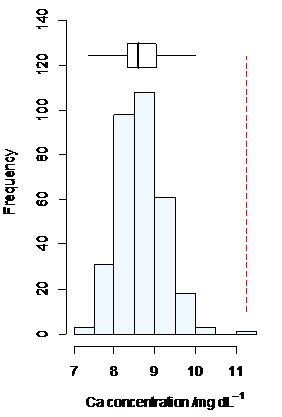

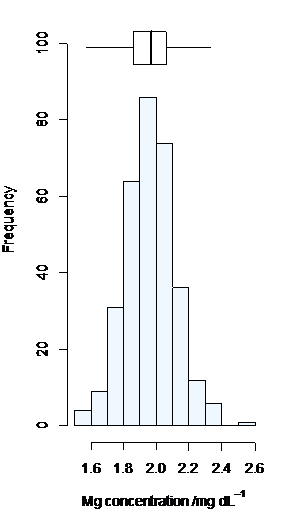


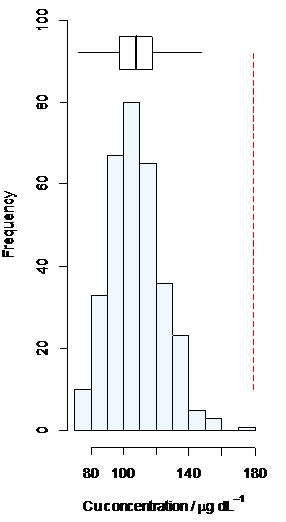

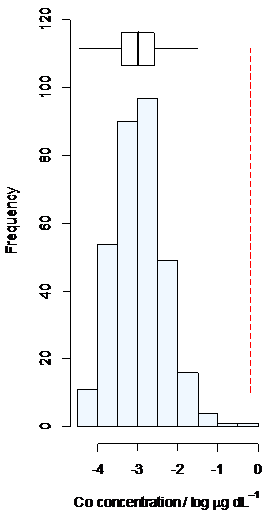


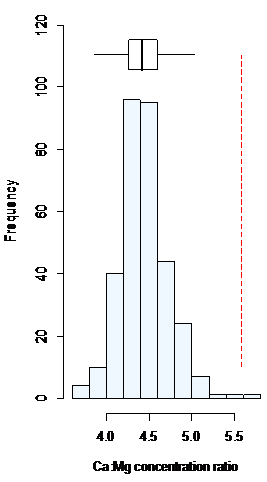

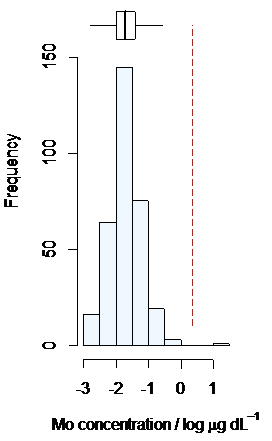

Supplement: Supplementary file 1 [file S0007114522000332sup.zip › S0007114522000319sup003.docx]
