# Supplementary material for: Effect of oat or rice flour on pulse-induced gastrointestinal symptoms and breath hydrogen in subjects sensitive to pulses and controls – a randomised cross-over trial with two parallel groups
Source: Br J Nutr. 2022 Jan 28;128(11):2181–92. doi: 10.1017/S0007114522000332 (PMC9661369; doi:10.1017/S0007114522000332)
Supplement: Supplementary file 1 [file S0007114522000332sup.zip › S0007114522000319sup001.doc]

**Supplementary Table 1**. Parameters of fitted variogram models and mean and median standardized squared prediction error. 95% interval for is 0.337 – 0.573.

| Trace metals | Estimator | c0 | c1 | φ /km |  |  | A_spatial_ | A_non-spatial_ |
| --- | --- | --- | --- | --- | --- | --- | --- | --- |
| Ca | Matheron | 0.146 | 0.210 | 201.8 | 0.975 | 0.347 | 2.2 | 34.4 |
|  | Cressie and Hawkins | 0.115 | 0.220 | 161.6 | 1.16 | 0.413 | 1.5 | 36.4 |
|  | Dowd | 0.085 | 0.247 | 130.0 | 1.41 | 0.458 | 26.4 | 47.2 |
| Mg | Matheron | 0.009 | 0.017 | 92.2 | 1.16 | 0.344 | −124.0 | −94.1 |
|  | Cressie and Hawkins | 0.007 | 0.018 | 71.4 | 1.28 | 0.397 | −115.4 | −92.5 |
|  | Dowd | 0.005 | 0.018 | 56.6 | 1.42 | 0.454 | −102.3 | −88.1 |
| Ca:Mg ratio | Matheron | 0.047 | 0.029 | 47.2 | 1.22 | 0.371 | −57.9 | −53.5 |
|  | Cressie and Hawkins | 0.039 | 0.033 | 48.2 | 1.41 | 0.448 | −66.5 | −56.8 |
|  | Dowd | 0.036 | 0.033 | 38.3 | 1.47 | 0.461 | −68.6 | −59.6 |
| Co | Matheron | 0.37 | 7.53 | 5e4 | — | — | 39.6 | 38.8 |
|  | Cressie and Hawkins | 0.34 | 0.07 | 95.0 | — | — | 39.7 | 37.4 |
|  | Dowd | 0.32 | 0.08 | 95.0 | — | — | 47.2 | 44.9 |
| Cu | Matheron | 251.7 | 15.1 | 87.2 | — | — | 330.6 | **327.3** |
|  | Cressie and Hawkins | 231.1 | 29.5 | 79.5 | — | — | 337.1 | **335.0** |
|  | Dowd | 223.6 | 32.8 | 101.4 | — | — | 339.3 | **337.7** |
| Mo | Matheron | 0.072 | 0.172 | 91.8 | 1.33 | 0.496 | −5.07 | 22.44 |
|  | Cressie and Hawkins | 0.078 | 0.166 | 126.2 | 1.35 | 0.515 | 4.95 | 26.32 |
|  | Dowd | 0.079 | 0.162 | 142.9 | 1.38 | 0.548 | 14.27 | 29.78 |
| Cu:Zn ratio | Matheron | 0.035 | 0 | — | — | — | −97.32 | −101.32 |
|  | Cressie and Hawkins | 0.033 | 0 | — | — | — | −102.84 | −106.84 |
|  | Dowd | 0.031 | 0 | — | — | — | −101.15 | −105.15 |

c0 is the nugget variance, c1 is the sill variance of the correlated structure, φ /km is the distance parameter of the exponential model, is the mean squared prediction error and is the mean squared deviation ratio.
